# Reduced microbial diversity in adult survivors of childhood acute lymphoblastic leukemia and microbial associations with increased immune activation

**DOI:** 10.1186/s40168-017-0250-1

**Published:** 2017-03-20

**Authors:** Ling Ling Chua, Reena Rajasuriar, Mohamad Shafiq Azanan, Noor Kamila Abdullah, Mei San Tang, Soo Ching Lee, Yin Ling Woo, Yvonne Ai Lian Lim, Hany Ariffin, P’ng Loke

**Affiliations:** 1 University Malaya Cancer Research Institute, University of Malaya, 50603 Kuala Lumpur, Malaysia; 2 Department of Pharmacy, Faculty of Medicine, University of Malaya, 50603 Kuala Lumpur, Malaysia; 3 Centre of Excellence for Research in AIDS (CERIA), University of Malaya, 50603 Kuala Lumpur, Malaysia; 4 The Peter Doherty Institute for Infection and Immunity, University of Melbourne, Melbourne, Australia; 5 Department of Pediatric, Faculty of Medicine, University of Malaya, 50603 Kuala Lumpur, Malaysia; 6 Departments of Microbiology and Medicine, New York University School of Medicine, New York, NY 10016 USA; 7 Department of Parasitology, Faculty of Medicine, University of Malaya, Kuala Lumpur, Malaysia; 8 Department of Obstetrics and Gynecology, Faculty of Medicine, University of Malaya, Kuala Lumpur, Malaysia

**Keywords:** Adult survivors of childhood cancer, Acute lymphoblastic leukemia, Microbiome, Alpha diversity, Microbiota dysbiosis, Immune activation, Inflammation

## Abstract

**Background:**

Adult survivors of childhood cancers such as acute lymphoblastic leukemia (ALL) have health problems that persist or develop years after cessation of therapy. These late effects include chronic inflammation-related comorbidities such as obesity and type 2 diabetes, but the underlying cause is poorly understood.

**Results:**

We compared the anal microbiota composition of adult survivors of childhood ALL (*N* = 73) with healthy control subjects (*N* = 61). We identified an altered community with reduced microbial diversity in cancer survivors, who also exhibit signs of immune dysregulation including increased T cell activation and chronic inflammation. The bacterial community among cancer survivors was enriched for *Actinobacteria* (e.g. genus *Corynebacterium*) and depleted of *Faecalibacterium*, correlating with plasma concentrations of IL-6 and CRP and HLA-DR+CD4+ and HLA-DR+CD8+ T cells, which are established markers of inflammation and immune activation.

**Conclusions:**

We demonstrated a relationship between microbial dysbiosis and immune dysregulation in adult ALL survivors. These observations suggest that interventions that could restore microbial diversity may ameliorate chronic inflammation and, consequently, development of late effects of childhood cancer survivors.

**Electronic supplementary material:**

The online version of this article (doi:10.1186/s40168-017-0250-1) contains supplementary material, which is available to authorized users.

## Background

Acute lymphoblastic leukemia (ALL) is the most common childhood cancer, which now has 5-year survival rates exceeding 80% as a result of improved therapy [1]. Adult survivors of childhood cancer have a higher risk of chronic comorbidities [2–4] and early mortality compared to their age-matched controls [5]. These comorbidities include diabetes mellitus [3, 6], metabolic syndrome, cardiovascular disease [7], renal insufficiency [3] and frailty [8]. The development of these conditions could be attributed to radiotherapy [9, 10] and chemotherapy [11], though the exact mechanisms remain unclear.

Chemotherapeutic agents have a broad impact on the gastrointestinal (GI) system including structural damage to the gut, dysregulation of the gut-associated lymphoid tissue [12] and alteration of the gut microbiota [13, 14]. Adult survivors of childhood cancer who received abdominal radiation, as well as exposure to anthracyclines and alkylating agents, report a higher incidence of GI tract complications [15]. In childhood myeloid leukemia, chemotherapy has been associated with a reduction in anaerobic bacteria and increased enterococci, persisting up to at least 6 weeks post treatment [13]. Multiple courses of broad-spectrum antibiotics for febrile neutropenia and antimicrobial prophylaxis [16, 17] may also have long-term effects on the gut microbiota.

The relationship between the gut microbiota and human health is now well-documented [18–20]. The imbalance or dysbiosis of microbial communities is known to be associated with the development of many different diseases, including diabetes mellitus, metabolic syndrome [21], atherosclerosis [22, 23] and frailty [24–26], all of which are also prevalent among childhood cancer survivors [3, 4]. It is difficult to determine whether dysbiosis is the cause or result of human disease [27], and causality has only been demonstrated in mouse models. In murine studies, dysbiosis can trigger systemic immune dysregulation through its local effects on T cells in the gut, as well as in the circulation [28–30]. The mechanisms by which microbes impact immune responses during homeostasis and disease is an area of intense investigation. Alterations in bacterial metabolites [31, 32] and increased intestinal translocation [33, 34] are mechanisms that have been proposed to link microbial dysbiosis with immune dysregulation. Specific alterations to the microbial communities are associated with markers of immune activation and chronic inflammation. Hence, monitoring gut microbial communities and inflammation status of ALL survivors may be a potential surveillance strategy for targeting health counseling. In addition, data on microbial communities may also be crucial for the design and testing of therapeutic interventions to restore microbial diversity thereby potentially reducing systemic inflammation.

We recently observed that a cohort of adult survivors of childhood leukemia in Malaysia exhibited signs of increased immune activation and chronic inflammation [35]. We hypothesized that this inflammatory phenotype could be associated with persistent changes in microbial communities. Using subjects from this cohort, we investigated whether adult survivors of childhood ALL have reduced microbial diversity compared to controls without history of cancers.

## Methods

### Study cohort

Participants for this study were selected from a larger study originally to explore the presence of a phenotype of aging on young adult leukemia survivors [35]. Adult survivors of childhood ALL who attended a late-effect surveillance clinic at the University of Malaya Medical Centre (UMMC), Malaysia, for their annual follow-up were recruited. Inclusion criteria were (1) individuals aged between 18 and 35 years, (2) at least 5 years since completion of leukemia treatment, (3) no history of bone marrow transplant and (4) no acute illness and not pregnant at the point of recruitment. Healthy controls who also fulfilled the inclusion criteria but without history of cancers were recruited among healthcare workers, siblings and volunteers. The study protocol was approved by the institutional ethical committees (reference number: MEC 2014/1093.65), and signed informed consent was obtained from all the participants for sample collection and data analysis.

### DNA extraction and 16S ribosomal RNA genes sequencing

Fecal microbiome sample was collected using sterile anal swabs and stored at −80 °C prior to processing. Anal swabs were more practical for collection in the clinic setting as opposed to obtaining a fecal sample. We assumed the microbiota obtained from anal swabs would resemble that of fecal samples as has been demonstrated in previous studies [36, 37].

DNA from anal swabs were extracted using the NucleoSpin® Tissue kit according to the manufacturer’s protocol (Macherey-Nagel, Germany). DNA was eluted with 50 μl of Buffer BE and stored at −20 °C. Subsequently, DNA library preparation and sequencing were performed in New York University Genomic Centre. Briefly, DNA samples were amplified for the variable 4 region (V4) of 16S rRNA gene using the method and primer constructs as previously described [38]. The forward primer construct contained the 5’ Illumina adapter, the forward primer pad, a two-base linker (‘GT’) and the 515F primer (5′-AAT GAT ACG GCG ACC ACC GAG ATC TAC ACT ATG GTA ATT GTG TGC CAG CMG CCG CGG TAA-3′). The reverse primer construct contained the 3’ Illumina adapter, a unique 12-base error-correcting Golay barcode, the reverse primer pad, a two-base linker sequence (‘CC’) and the 806R primer (5′-CAA GCA GAA GAC GGC ATA CGA GAT NNN NNN NNN NNN AGT CAG TCA GCC GGA CTA CHV GGG TWT CTA AT-3′). 16S rRNA V4-targeted polymerase chain reaction (PCR) was carried out in triplicate with thermal cycling of 94 °C for 3 min, followed by 35 cycles of 94 °C for 45 s, 50 °C for 60 s, 72 °C for 90 s, and lastly, 10 min at 72 °C to confirm full amplification. Three replicate amplicons were measured using TapeStation for DNA concentrations and pooled at equimolar ratio. Sequencing on the amplicons was conducted on the Illumina MiSeq system (Illumina, San Diego CA, USA).

### 16S rRNA gene sequences analysis

The paired-end sequencing reads were joined using the fastq-join function from EA-utils with default parameters followed by demultiplexing using the 12-base Golay barcodes. Demultiplexing was done with the default quality-filtering parameters: minimum quality score of 25, minimum/maximum sequence length of 200/1000, no ambiguous bases and no mismatches in the primer sequence [39]. Sequences were then analyzed with QIIME version 1.8.0 [40]. Using *pick_open_reference_otus.py* workflow, operational taxonomic unit (OTU) picking was performed first, with a closed reference method by aligning the sequences to reference in Greengenes 13.8 database. Unaligned sequences were clustered by de novo method using the UCLUST consensus taxonomy assigner, at the minimum of 97% sequence similarity.

After picking for OTUs, samples with low reads (<1000) were removed using *filter_samples_from_otu_table.py* script. We had on average 7313 reads per sample for 134 samples (a total of 980,062 reads, ranging from 1127 to 28,299 reads). After that, OTUs were grouped at different levels of taxonomy classification (phylum, class, order, family and genus) and normalized at each level to get the relative abundance of each taxonomy using *summarize_taxa_through_plots.py* script.

### Microbial diversity analysis

OTU-based alpha diversity was estimated by calculating three diversity matrices, phylogenetic distance, observed OTUs and Chao1 using QIIME workflow (*alpha_rarefaction.py*). First, rarefaction analysis was iterated over 10 depths up to the 1000 reads depth (to match the minimum sampling depth) with 10 times subsampling at each depth. Rarefaction curves were generated for each matrix. Non-parametric test was used to compare the statistical significance of the rarefraction curves between the survivor and control groups implemented in QIIME function (*compare_alpha_diversity.py*) as the data distribution was not normal. Chao1 index, phylogenetic distance and number of observed OTUs at the rarefaction of 1000 reads were compared.

### Inferred metagenomics and functions using PICRUSt

Closed-reference OTU table was generated using QIIME script *pick_closed_reference_otus.py* for community metagenome functions inference. First, the original 16S rRNA sequencing data was quality-filtered and demultiplexed, followed by taxonomy assignment of the representative sequences to the reference sequences from Greengenes 13.8 database at 97% similarity. The resulting closed-reference OTU table was passing through the PICRUSt workflow on the online Galaxy interface from the Huttenhower Lab (https://huttenhower.sph.harvard.edu/galaxy/). Each OTU was first normalized for 16S rRNA copy number, followed by metagenome prediction. Functional annotation and pathway inference of the metagenome was done using Kyoto Encyclopedia of Genes and Genomes (KEGG) database at pathways of hierarchy levels 2 and 3 [41]. Linear discriminant analysis (LDA) Effect Size (LEfSe) was used to identify the differentially abundant KEGG pathways in the survivor and control groups.

### LDA Effect Size analysis

To identify the bacterial taxa and the predicted KEGG functional pathways that are differentially abundant in the survivors’ and controls’ microbiome, LEfSe analysis was performed using the online Galaxy interface (http://huttenhower.sph.harvard.edu/galaxy/). LEfSe uses the non-parametric factorial Kruskal-Wallis rank-sum test to detect features that are significantly different in abundance between the survivor and control groups. The effect sizes of the identified features were then estimated with linear discriminant analysis model [42]. False discovery rates of the resulting *p* values were corrected using the *p.adjust()* function with the Benjamini-Hochberg algorithm implemented in R, and reported as *q* values (also summarized in Additional file 1: Table S1).

### Group associated with OTU selection using sparse partial linear square discriminant analysis (sPLS-DA)

sPLS-DA [43] was conducted to select the most discriminative OTUs associated with survivor or control groups. OTU data was added with a single pseudocount prior to compositionally normalization using total sum scaling (TSS) and centered log-ratio (CLR) transformation. sPLS-DA analysis was then performed using the *splsda()* function to select 10 features (OTUs) each on the first and second principal components respectively to best discriminate survivor group or control group. Individual samples were presented on a PCA plot based on the selected OTUs and are distinguished by group with color and 95% confidence eclipses using the *plotIndiv()* function. The contribution of the OTUs that were associated to each group on the first and second components are presented with contribution plots using the *plotLoadings()* function. The abundance of each selected OTUs are presented on clustering heatmaps using the *cim()* function. The taxa hierarchy of each OTU is summarized in Additional file 1: Table S2. All the analyses were conducted using mixOmics package version 6.1 implemented in R software [43].

### Cytokine and inflammatory markers

Peripheral blood was collected from participants in EDTA vacutainer and processed within 4 h of sampling as previously described [44]. Plasma IL-6 was measured by enzyme-linked immunosorbent assay (ELISA) using the Quantikine HS IL-6 kit (R&D Systems, Minneapolis, USA). All assays were conducted in duplicate and according to manufacturer’s instructions. Plasma CRP was measured by the hospital central diagnostic laboratory.

### T cells activation markers

T cells activation was assessed with the expression of HLA-DR using flow cytometry and reported as %HLA-DR+ CD4 or CD8 T cells. Immunophenotyping was performed on peripheral whole blood as previously described [35]. Briefly, 100 μl of blood was stained for surface markers CD3-PerCp-Cy5.5 (clone SK7), CD4-PE-Cy7 (clone SK3), CD8-APC-H7 (clone SK1) and HLA-DR-BV421 (clone G46-6) using antibody cocktails for 15 min in the dark. Red blood cells were then lysed using a 1:10 dilution of BD lysis buffer (all antibodies and reagents are from BD Pharmigen, San Jose, CA) for 10 min, and the cells were washed once with phosphate-buffered saline (PBS). Samples were acquired on a BD FACSCanto II (BD Biosciences, San Jose, CA) for 100,000 events and analyzed using the FACS Diva software (version 6.0). The gating strategy used is shown in Additional file 1: Figure S1.

### Statistical analysis

Statistical analysis was performed using SPSS software (version 21, IBM) and R software (version 3.3.0). Data was checked with Shapiro-Wilk test for distribution prior to significance tests. Unpaired two-tailed *t* tests (Student’s *t* test for normally distributed data and Mann-Whitney test for skewed data) were used to evaluate differences between two groups. Correlation of each taxa feature identified from LEfSe analysis with immunological markers were estimated with Spearman rank partial correlation test adjusted for age, gender and BMI using the *pcor.test()* function in the ppcor package implemented in R [45].Correlation analysis was performed on the combined data (survivors and controls), survivor only group and control only group, to show if the correlation for all of the other taxa of interest is observed when data from both or either survivors or controls are used separately. Correlation coefficient (*rho*) and *p* value are presented in Additional file 1: Table S3.

## Results

### Adult survivors of childhood ALL have reduced anal microbial diversity

Anal swab samples were collected from 73 adult survivors of childhood ALL and 61 healthy controls (see Table 1). The demographic characteristics of survivors and controls were comparable. We noted a slightly higher median age in survivors (26 years, interquartile range (IQR) 22–29.5) compared to that in controls (23 years (IQR 21–24)). For the survivors, median age at diagnosis was 5 years (IQR 2.25–9) and median duration since treatment cessation was 18.5 years (IQR 14–23). The clinical and demographic characteristics of the study participants are summarized in Table 1.

**Table 1:** Participant clinical and demographic characteristics

	Group		*p* value
	Survivors	Controls	
Number in each group	*N* = 73	*N* = 61	
Sex, *n* (%)			0.378Chi
Male	28 (38.4%)	28 (45.9%)	
Female	45 (61.6%)	33 (54.1%)	
Ethnicity			0.159Chi
Malay	23 (31.5%)	21 (34.4%)	
Chinese	45 (61.6%)	29 (47.5%)	
Indian	5 (6.8%)	10 (16.4%)	
Others	0	1 (1.6%)	
Age at recruitment, years (IQR)	26 (22–29.5)	23 (21–24)	0.001MW
Age at diagnosis, years (IQR)	5 (2.25–9)	–	
Diagnosis, *n* (%)			–
ALL	73 (100%)	–	
Duration of cancer therapy, years (IQR)	18.5 (14–23)	–	
Cancer therapy history		–	
Anthracyclines, *n* (%)	53 (73.6%)	–	
Alkylating agents, *n* (%)	55 (76.4%)	–	
Anthracylines and alkylating agents, *n* (%)	52 (72.2%)	–	
Radiotherapy received, *n* (%)	36 (50%)	–	
Cancer relapse, *n* (%)	2 (2.7%)		
Second neoplasms, *n* (%)	3 (4.1%)	–	
Diabetes mellitus or hypertension, *n* (%)	3 (4.1%)	0	
BMI, kg/m^2^ (IQR)	23.5 (20.7–27.5)	21.8 (20.3–25.2)	0.006T
Mode of birth (caesarean/vaginal), *n*/*n*	5/63	9/50	0.238Chi
^a^Antibiotic intake, *n* (%)	7 (9.6%)	7 (11.5%)	0.789Chi

Data shown are median (interquartile range, IQR) or *n* (%). *n* = number of subjects. Variables are significantly different between survivors compared to controls if *p* < 0.05 on *T* = Student’s *t* test, *MW* = Mann Whitney test or *Chi* = Chi-square tests

^a^Number of subjects who consumed any antibiotic within 1 month prior to recruitment

**Fig. 1:**
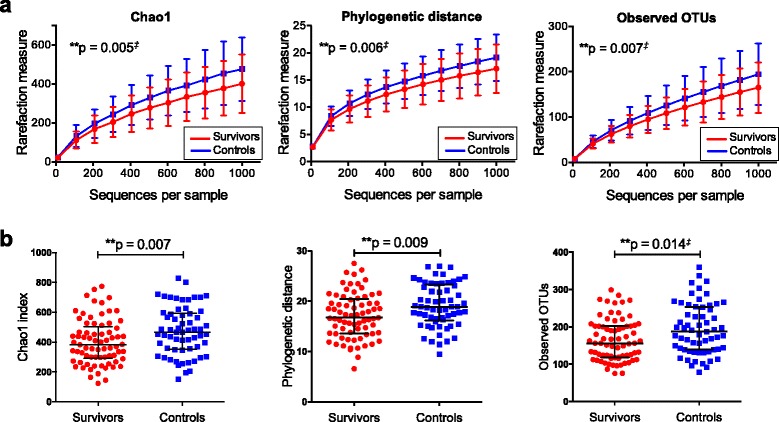
Reduced microbial diversity in adult survivors of childhood ALL. Rarefaction curves for alpha diversity measured with three different matrices: Chao1, phylogenetic distance and observed OTUs. Microbial diversity was reduced among the survivors as compared to controls (**a**). Number of observed OTUs, Chao1 index and phylogenetic distance (at the rarefaction of 1000 reads) was reduced in the survivor group as compared to that in controls (**b**). Data shown as median ± interquartile range and *p* values was calculated with Student’s *t* test or Mann-Whitney test. *Double dagger* denotes non-parametric test was used. *OTUs* operational taxonomic units

Despite being asymptomatic, the adult survivors of childhood ALL had significantly reduced microbial diversity compared to the controls as determined by measurements of alpha-diversity. Estimations with three different alpha diversity matrices (Chao1, observed OTUs and phylogenetic distance) consistently showed significantly reduced diversity (*p* ≤ 0.01) for the survivor group compared to the control group (Fig. 1a, b). Reduced microbial diversity is often associated with chronic diseases [46–48].

### A different microbial signature in adult survivors of childhood ALL

**Fig. 2:**
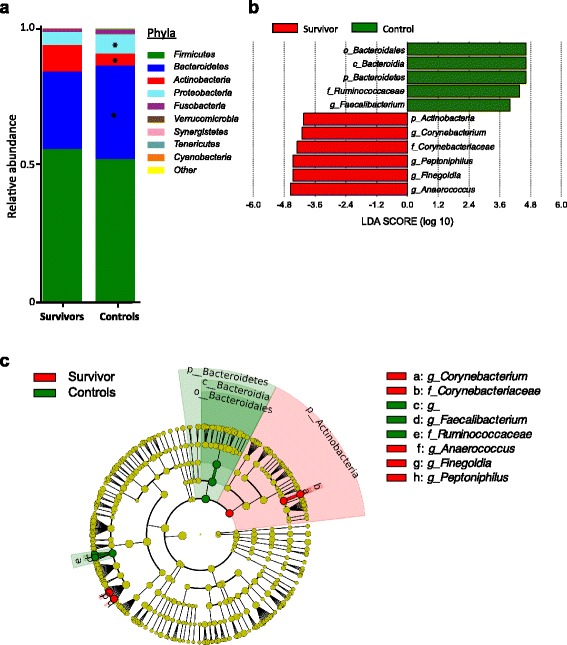
Alterations to microbial communities in adult survivors of childhood ALL. Averaged relative abundances of the 10 most abundant phyla in the survivor and control group. Phyla *Bacteroidetes* and *Proteobacteria* were reduced, while *Actinobacteria* was enriched in survivors (*asterisk* denotes *p* < 0.05, measured with Mann-Whitney test) (**a**). We identified taxa (at taxonomic levels: phylum, class, order, family and genus) that were differentially abundant between survivors and controls using linear discriminant analysis (LDA) Effect Size (LEfSe) analysis, at a LDA score >4 and FDR-adjusted *q* value ≤0.05 (**b**). Taxonomic cladogram was also generated using LEfSe analysis of the taxa data (**c**)

The dominant phyla in both groups are the *Firmicutes* and *Bacteroidetes* (Fig. 2a and Additional file 1: Figure S2a), consistent with previous findings that microbial composition of anal swab samples closely resemble fecal samples [36]. Survivors and controls did not cluster into clear distinct groups with beta-diversity estimates (Additional file 1: Figure S2b). However, supervised comparison between the two groups by LEfSE analysis [42] and after correction with FDR adjustment identified several differentially abundant microbial taxa between the survivor and control groups (Fig. 2b, c, Additional file 1: Table S1). At the phylum level, the survivors’ microbiota community was slightly enriched for *Actinobacteria* while depleted of phylum *Bacteroidetes* and *Proteobacteria* (Fig. 2a). Although relative abundance of the phyla *Firmicutes* is not significantly different between groups, specific members of *Firmicutes* were enriched (*Tissierellaceae* and *Staphylococaceae*), while others were reduced (*Ruminococaceae* and *Lachnospiraceae*) in the survivor group. Also of interest is the reduced relative abundance of *Faecalicabacterium.* LDA score, *p* value and FDR-corrected *q* value of the significantly differently abundant taxa are summarized in Additional file 1: Table S1. In this study, we show that the microbial composition among ALL survivors exhibit abundant differences in comparison to healthy controls, indicating a potential microbial signature for these survivors. Of particular interest, the bacterial taxa, such as *Faecalicabacterium*, *Ruminococaceae* and *Lachnospiraceae* that are known to be reduced in abundance for other chronic diseases (for example, inflammatory bowel disease and psoriatic arthritis) [47, 49], were also less abundant among the cancer survivors. Therefore, adult survivors of childhood ALL share some features of microbial dysbiosis with individuals with chronic diseases [50, 51] despite being otherwise asymptomatic.

**Fig. 3:**
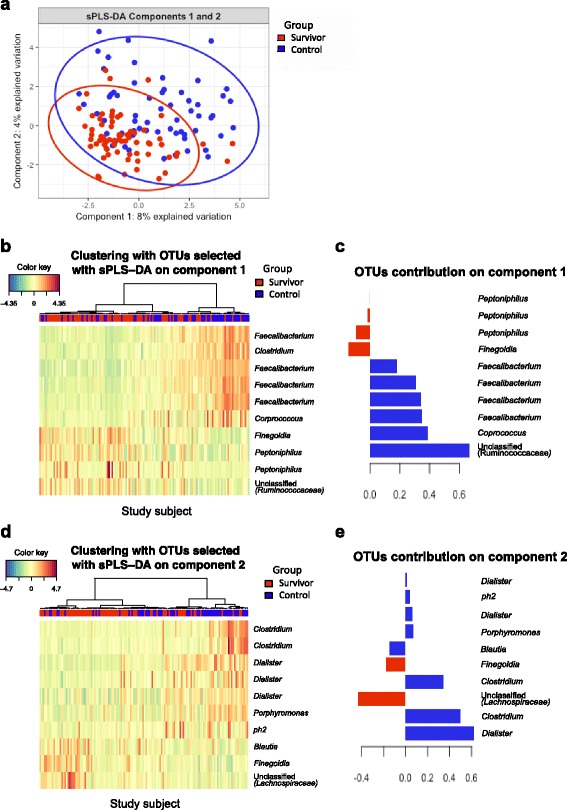
Identification of groups (survivors or controls) associated with OTUs using CLR transformation and sPLS-DA model. Individual samples were presented on a PCA plot based on the selected OTUs and are distinguished by group with color and 95% confidence eclipses (**a**). The abundance of each selected OTUs are presented on clustering heatmaps (**b**, **d**). The contribution of the OTUs that associated to each group on the first and second components are presented with contribution plots (**c**, **e**)

We next utilized an alternative approach to further reduce potential noise and compositional artifacts in the dataset, by using a sparse (to ensure selection of relatively few OTUs) partial least squares discriminant analysis (sPLS-DA) to identify specific OTUs that could potentially distinguish between the cancer survivors from controls. With a threshold set at 10 OTUs, we observed comparable separation of survivors and controls as sPLS-DA analysis with larger number of OTUs (Additional file 1: Figure S3 (show heatmaps of 20 OTUs, 100 OTUs, and optimal number of OTUs selected with the MixOmics *tune.splsda()* process)). However, there is still considerable overlap between survivors and controls (Fig. 3a, b, d, Additional file 1: Figure S3). Notably, the OTUs identified to contribute towards component 1 (8% variance explained) and component 2 (4% variance explained) predominantly belong to taxa such as *Faecalibacterium*, *Finegoldia* and *Peptoniphilus* that were previously identified using LEfSe analysis (Fig. 3c, e). Details of taxa hierarchy and contribution score for each of the OTUs selected with sPLS-DA are summarized in Additional file 1: Table S2. Overall, consistent differences were observed between survivors and controls for specific components of the microbiota, but this was insufficient to clearly distinguish survivors from controls.

### T cell activation and systemic inflammation markers are associated with dysregulated microbial taxa

**Fig. 4:**
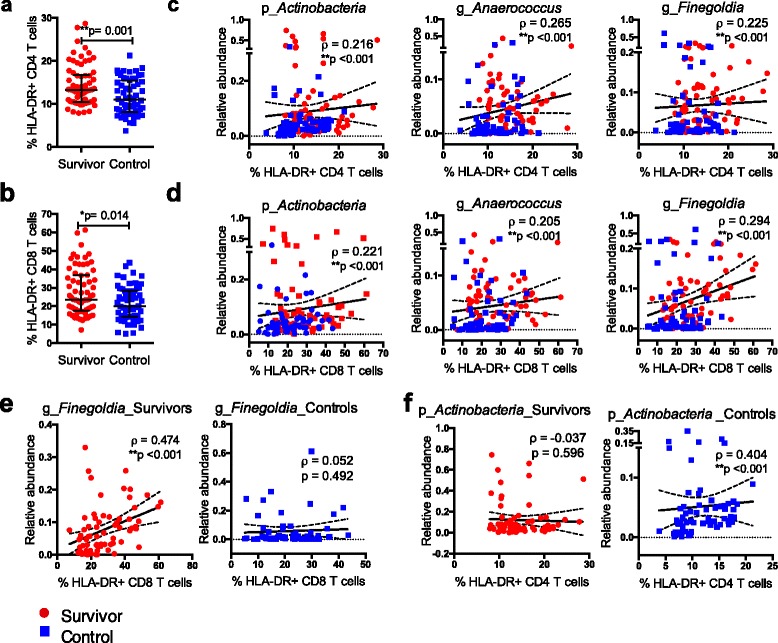
Increased T cell activation is associated with alterations to microbial communities. Both the levels of CD4 and CD8 T cell activation were higher in the survivors than in controls (**a**, **b**). Relative abundances of specific taxa identified from LEfSe analysis were associated with %HLA-DR+ CD4 and CD8 T cells respectively (**c**, **d**). Relative abundance of genus *Finegoldia* was positively correlated with %HLA-DR+ CD8 T cells among the survivors, while the same association was not observed in the control group (**e**). In contrast, relative abundance of phylum *Actinobacteria* was positively correlated with %HLA-DR+ CD4 T cells among the controls, while the same association was not observed in the survivor group (**f**). Data shown as median ± interquartile range and *p* values was calculated with Mann-Whitney test for group comparison. Data shown as correlation coefficient (*ρ*) and confounders-adjusted *p* value calculated with *Spearman* rank partial correlation test adjusted for age, gender and BMI for the correlation between taxa and immune markers. Linear regression line was fitted for visualization purposes only. *Red circles* denote the survivors, while *blue squares* denote the controls. *p_* = phylum; *g_* = genus

The T cells of the immune system are heavily influenced by the microbiota and play an important role in homeostasis [52, 53]. It has been previously reported that young adult leukemia survivors show increased immune activation [35]. Immune activation quantified by the expression of HLA-DR on CD4+ and CD8+ T cells was correlated to the anal microbiota composition. We first demonstrated that the ALL survivors exhibited increased activation (%HLA-DR) of CD4+ and CD8+ cells, in comparison to controls (Fig. 4a, b), which is consistent with our previous report [35]. We then asked whether the activation status of the T cells was associated with the observed differences in microbial taxa between the survivor and control groups after adjustment for age, gender and BMI. We found that there was indeed a significant positive correlation between relative abundance values of the phyla *Actinobacteria* and the frequency of HLA-DR+CD4+ cells and HLA-DR+CD8+ cells (Fig. 4c, d, Additional file 1: Table S3). More specifically, two genera in the *Tissierellaceae* family, *Aneerococcus* and *Finegoldia*, were positively associated with activation of CD4+ T cells (Fig. 4c, Additional file 1: Table S3) and CD8+ T cells (Fig. 4d, Additional file 1: Table S3). Interestingly, relative abundance of *Finegoldia* was predominantly associated with CD8+ T cell activation among the survivors (rho = 0.474, *p* < 0.001), but there was no association in the control group when we analyzed the groups separately (Fig. 4e, Additional file 1: Table S3). In contrast, relative abundance of *Actinobacteria* was predominantly associated with CD4+ T cell activation among the controls (rho = 0.404, *p* < 0.001), but there was no association in the survival group when we analyzed the groups separately (Fig. 4f, Additional file 1: Table S3). This raises the possibility that an elevated abundance of *Finegoldia* may specifically drive higher CD8+ T cell activation among the survivors while *Actinobacteria* abundance is associated with CD4 T cell activation among controls but not among the survivors. The implications of these associations are unclear and would require further experimentation to demonstrate causality.

**Fig. 5:**
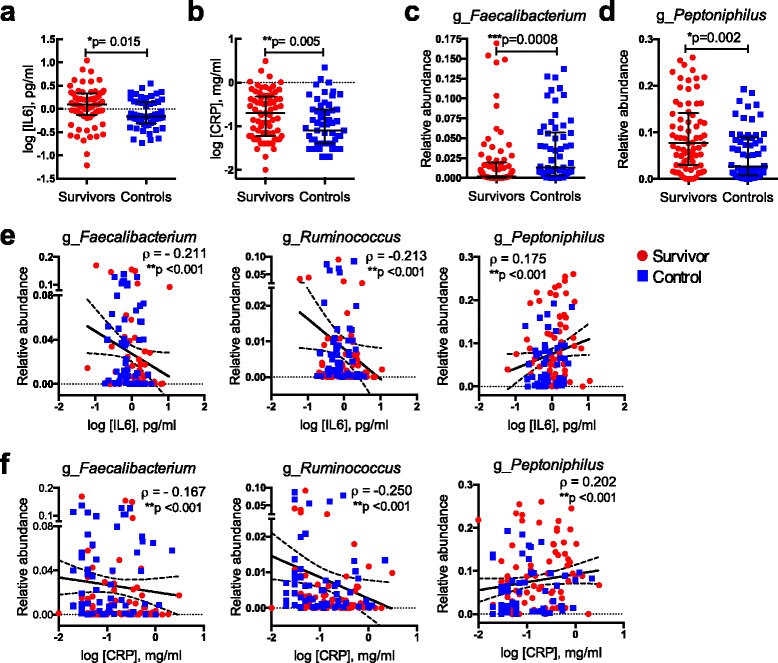
Systemic inflammatory markers are associated with variation in microbial communities. The levels of both systemic inflammatory markers plasma IL-6 and CRP were higher in the survivors than in controls (**a**, **b**). Microbial community in the survivors was reduced with *Faecalibacterium* but enriched for *Peptinophilus* (**c**, **d**)*.* Both IL-6 and CRP were negatively associated with *Faecalibacterium* and *Ruminococcus*, while positively associated with *Peptoniphilus* (**e**, **f**). Data shown as median ± interquartile range and *p* values was calculated with Mann-Whitney test for group comparison. Data shown as correlation coefficient (*ρ*) and confounders-adjusted *p* value calculated with *Spearman* rank partial correlation test adjusted for age, gender and BMI for the correlation between taxa and immune markers. Linear regression line was fitted for visualization purposes only. *Red circles* denote survivors, while *blue squares* denote controls. *g_* = genus

In addition to increased activation of T cells, we found that some biomarkers of inflammation, namely, circulating levels of interleukin (IL)-6 and C-reactive protein (CRP) as determined by ELISA, were elevated in the adult survivors of childhood ALL compared to controls (Fig. 5a, b). Notably, IL-6 and CRP measurements are negatively associated with the relative abundance values of *Faecalibacterium* and *Ruminococcus*, while being positively associated with *Peptoniphilus* (Fig. 5e, f, Additional file 1: Table S3)*.* Changes in abundance of *Faecalibacterium prausnitzii* in particular has been linked with dysbiosis in several human diseases (e.g. reduced in inflammatory bowel disease [49, 54] and ulcerative colitis [50]). Furthermore, *F. praunitzii* has been shown to induce IL-10 secretion, which is an anti-inflammatory cytokine [55]. The reduced abundance of *F. praunitzii* and its association with the increase inflammatory biomarkers may be significant for the childhood ALL survivors; however, demonstration of a causal relationship would require further experimentation.

### Functional differences in bacterial communities among adult survivors of childhood ALL

**Fig. 6:**
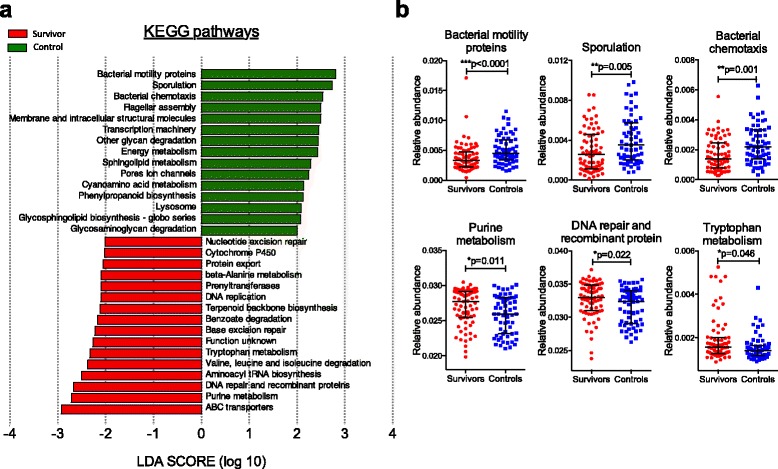
Alterations to the functional profiles of microbial communities in adult survivors of childhood ALL by inferred metagenomics. Functional pathways were inferred from OTUs using PICRUSt and annotated with KEGG database. We identified the differently abundant pathways with LEfSe (at LDA threshold of 2) and plotted the LDA scores of pathways of hierarchy level 3 (**a**). Genes related to the pathways of purine metabolism, DNA repair and recombinant protein and tryptophan metabolism were significantly more abundant in survivors’ microbiome. In contrast, pathways related to bacterial cellular processes and signaling including bacterial motility proteins, sporulation and bacterial chemotaxis were significantly more abundant in controls’ microbiome (**b**). Data shown as median ± interquartile range and *p* values was calculated with Mann-Whitney test

We next utilized inferred metagenomic analysis [41] to determine if there are specific alterations in metabolic pathways encoded by bacterial communities between the survivor and control groups (Fig. 6a). Interestingly, the bacterial microbiome of the survivors are enriched for several inferred amino acid pathways, including purine metabolism and tryptophan metabolism, as well as for DNA repair and recombinant proteins (Fig. 6b). Tryptophan metabolism in particular is of significant interest because it has been associated with microbial dysbiosis in HIV-infected patients [56]. The inferred pathways that are reduced in abundance in the survivors’ microbial communities encode for bacterial motility proteins, sporulation and bacterial chemotaxis. These results indicate that in addition to community differences, there may be differences to the functionalities of the microbiome between survivors and controls.

## Discussion

Our results provide the first evidence that asymptomatic adult survivors of childhood ALL have indications of microbial dysbiosis that share characteristics with alterations previously associated with the risk of immunological and metabolic diseases, including obesity. Additionally, there are specific relationships between the altered bacterial taxa and biomarkers of T cell activation and systemic inflammation, raising the hypothesis that differences in the bacterial communities may be associated with dysregulated immune activation. There is increasing evidence that environmentally induced alterations to the microbiota can be irreversible [57, 58]. Here, we also found that dysbiosis can be detected and probably persist for years after successful completion of cancer therapy.

Of particular significance is the reduction in microbial diversity among the adult survivors of childhood ALL compared to controls. Such clear differences in alpha diversity between groups (with a relatively small sample size) are rarely observed between healthy, or even diseased individuals (e.g. [59]), unless they are suffering from intestinal diseases such as inflammatory bowel disease [60, 61]. Microbial diversity increases and stabilizes over the first 3 years of life [58]; hence, dysbiosis during childhood is more likely to persist. It is now well documented that birth by caesarean section [62], obesity and antibiotic use are correlated with reduced microbial diversity, and reduced diversity during early life can be associated with inflammatory diseases such as allergic diseases [63], and children at high risk of type 1 diabetes have decreased microbial diversity over time [64].

Although there are significant differences in age and BMI (Table 1 and Additional file 1: Figure S4) between the survivors and controls, we corrected for potential confounding effects of age, BMI and gender with partial correlation analysis (as described in methodology section), and the relationship between bacterial taxa and immune activation markers remain significant. Colonic transit time is another potential confounder [65] that we did not measure in our study participants. We also did not assess incidences of GI complications, which has been reported to be greater in the adult survivors of childhood cancer [15] and could be associated with microbiome diversity and composition. Prolonged treatment with antibiotics during cancer therapy may contribute to microbial dysbiosis. All the childhood ALL survivors in this cohort received cotrimoxazole throughout the 2-year course of cancer therapy as prophylaxis against *Pneumocystis jerovici*; however, gut decontamination with quinolones was not practiced. Future studies with longitudinal samples from childhood ALL survivors are crucial to determine if this reduction in microbial diversity is a result of radiation and chemotherapy and persists into adulthood. Data from a few ALL survivors (*N* = 19) that are of a younger age (9–17 years old) indicates this reduced microbial diversity is present prior to adulthood (Additional file 1: Figure S5).

We also noted with interest that relative abundance values of the genus *Faecalibacterium* were reduced among the adult survivors of childhood ALL and negatively associated with the inflammatory biomarkers IL-6 and CRP. *F. prausnitzii* is one of the most abundant components of the gut microbiota [66]. For inflammatory bowel disease patients in particular, there are multiple studies showing differences in abundance for *Faecalibacterium* relative to healthy controls [50, 51, 55]. Additionally, a reduced abundance of *F. pausnitzii* has also been associated with the frailty index in a recent large population-based study [26], a phenotype previously described to be prevalent among young adult survivors of childhood cancer [8]. As a producer of short-chain fatty acids (SCFA) such as butyrate [67], it may be linked to regulation of intestinal inflammation through increasing regulatory T cells [68]. Future studies, including longitudinal studies, should monitor the abundance of *F. prausnitzii* in the feces of young adult survivors of childhood ALL as an indicator of intestinal health. A reduction in *F. prausnitzii* abundance was also observed in children with acute myeloid leukemia during treatment and 6 weeks post treatment [13], indicating that the dysbiosis we find here may have persisted years after cessation of chemotherapy.

Because of the limited quantities of microbial DNA available, it was not possible to perform complete metagenomic analyses of the participant samples. We instead used an inferred metagenomic approach to generate a preliminary overview of differences in microbiome functionality between the survivors of childhood ALL and controls. We noticed that tryptophan metabolism is higher among the survivors. Increase in tryptophan metabolism has also been previously reported in HIV-infected patients, and the higher level of tryptophan catabolism correlates with markers of chronic immune activation and inflammation [56]. While we have not measured production of kynurenine in this study, circulating levels of the inflammatory cytokine IL-6 have been shown to correlate with kynurenine production in HIV patients. The interferon-inducible enzyme indoleamine 2,3-dioxygenase 1 (IDO1) is associated with intestinal lymphoid tissue disruption, depletion of TH17 cells and chronic inflammation [69]. Consistent with the HIV study, we find here that bacterial taxa enriched among the survivors of childhood ALL may encode for enzymes that performs the same catabolic function as human IDO1, raising the hypothesis that the survivors may bear similarities in the mechanism of intestinal disruption that was previously described in HIV-infected patients [69, 70]. While interesting, these observations are based on inferred function with 16S sequences only and hence highly speculative. The microbially derived tryptophan catabolites and their effects on the host are considerably diverse [71, 72]. Future studies quantifying trytophan catabolites (such as kynurenine) will help determine the relationship between microbial changes in the childhood cancer survivors and tryptophan metabolism.

## Conclusions

While the mechanisms underlying our observed associations here are still poorly understood, the basic observation of dysbiosis and reduced microbial diversity in adult survivors of childhood ALL raises several crucial hypotheses. The composition of certain members of the microbial communities are associated with biomarkers of immune activation, namely, the higher levels of T cell activation and circulating levels of IL-6 and CRP. Hence, community dysbiosis may be driving low-grade inflammation, although, to establish causation, additional functional studies (e.g. transfer of microbiota samples into germ-free animals) would be required. More importantly, our findings here raise the possibility that therapeutic interventions that could restore microbial diversity and reverse dysbiosis to the ALL survivors may help to mitigate long-term effects. While we still do not know how to restore a healthy microbial community in people, multiple approaches (e.g. probiotics [73]), fecal transplantation [74] and helminth colonization [75, 76] are being investigated, which may be applicable to ALL survivors in the future. It is also important to note that in this study, we have not demonstrated a causal relationship between the altered microbial taxa and chronic inflammation. Future work to establish causality would involve isolation of specific taxa and transferring of anaerobically cultured clones into germ-free animals and demonstrating an increased inflammatory response in the recipient animals.

## Additional files

Additional file 1: Table S1. Bacterial taxa are different in abundance between survivors and controls, after Benjamini-Hochberg correction. **Table S2.** Taxonomic hierarchy and relative contribution of the sPLS-DA identified group-specific OTUs in the first and second component. **Table S3.** Partial correlation test, controlling for age, gender and BMI. **Figure S1.** Immunophenotyping of T cell activation. **Figure S2.** Taxonomy plot and beta diversity. **Figure S3.** Identification of groups (survivors or controls) associated with OTUs using CLR transformation and sPLS-DA model with a threshold set at a different number of OTUs. **Figure S4.** Body mass index (BMI) between adult survivors of childhood ALL and controls. **Figure S5.** Reduced microbial diversity in young (9–17 years old) survivors of childhood ALL. (PDF 22117 kb)

## Abbreviations

ALL: Acute lymphoblastic leukemia
CLR: Centered log-ratio
CRP: C-reactive protein
FDR: False discovery rate
IL: Interleukin
IQR: Interquartile range
KEGG: Kyoto Encyclopedia of Genes and Genomes
LDA: Linear Discriminant Analysis
LEfSe: Linear discriminant analysis (LDA) Effect Size
OTU: Operational taxonomic unit
sPLS-DA: Sparse partial least squares discriminate analysis
TTS: Total sum scaling
