# Supplementary material for: Reduced microbial diversity in adult survivors of childhood acute lymphoblastic leukemia and microbial associations with increased immune activation
Source: Microbiome. 2017 Mar 20;5:35. doi: 10.1186/s40168-017-0250-1 (PMC5359958; doi:10.1186/s40168-017-0250-1)
Supplement: Additional file 1: Table S1. — Bacterial taxa are different in abundance between survivors and controls, after Benjamini-Hochberg correction. Table S2. Taxonomic hierarchy and relative contribution of the sPLS-DA identified group-specific OTUs in the first and second component. Table S3. Partial correlation test, controlling for age, gender and BMI. Figure S1. Immunophenotyping of T cell activation. Figure S2. Taxonomy plot and beta diversity. Figure S3. Identification of groups (survivors or controls) associated with OTUs using CLR transformation and sPLS-DA model with a threshold set at a different number of OTUs. Figure S4. Body mass index (BMI) between adult survivors of childhood ALL and controls. Figure S5. Reduced microbial diversity in young (9–17 years old) survivors of childhood ALL. (PDF 22117 kb) [file 40168_2017_250_MOESM1_ESM.pdf]

Additional file 1

Reduced Microbial Diversity In Adult Survivors of Childhood Acute Lymphoblastic Leukemia Is Associated With Increased Immune Activation.

Ling Ling Chua[1§], Reena Rajasuriar[2,3,4§]*. Mohamad Shafiq Azanan[1,5], Noor Kamila Abdullah[3], Mei San Tang[6], Soo Ching Lee[3,7], Yin Ling Woo[1,3,8], Yvonne A.L. Lim[3,7], Hany Ariffin[1,5]*, P'ng Loke[6]*.

Table S1: Bacteria taxa different in abundance between survivors and controls, after Benjamini-Hochberg correction.

Taxonomic Hierarchy	Enriched in Group	log LDA score	p-value	q-value
Bacteria|p_Bacteroidetes|c_Bacteroidia|o_Bacteroidales	Control	4.6180	0.0023	0.0456
Bacteria|p_Bacteroidetes|c_Bacteroidia	Control	4.6180	0.0023	0.0456
Bacteria|p_Bacteroidetes	Control	4.6141	0.0025	0.0456
Bacteria|p_Actinobacteria	CCS	4.5531	0.0018	0.0456
Bacteria|p_Actinobacteria|c_Actinobacteria|o_Actinomycetales|f_Corynebacteriaceae|g_Corynebacterium	CCS	4.4601	0.0024	0.0456
Bacteria|p_Actinobacteria|c_Actinobacteria|o_Actinomycetales|f_Corynebacteriaceae	CCS	4.4601	0.0024	0.0456
Bacteria|p_Firmicutes|c_Clostridia|o_Clostridiales|f_Ruminococcaceae	Control	4.3606	0.0008	0.0397
Bacteria|p_Firmicutes|c_Clostridia|o_Clostridiales|f__Tissierellaceae_|g_Peptoniphilus	CCS	4.2978	0.0016	0.0456
Bacteria|p_Firmicutes|c_Clostridia|o_Clostridiales|f__Tissierellaceae_|g_Finegoldia	CCS	4.0998	0.0004	0.0397
Bacteria|p_Firmicutes|c_Clostridia|o_Clostridiales|f__Tissierellaceae_|g_Anaerococcus	CCS	4.0416	0.0003	0.0397
Bacteria|p_Firmicutes|c_Clostridia|o_Clostridiales|f_Ruminococcaceae|g_Faecalibacterium	Control	4.0090	0.0008	0.0397
*Bacteria|p_Firmicutes|c_Clostridia|o_Clostridiales|f_Ruminococcaceae|g_*	Control	3.9992	0.0012	0.0456
Bacteria|p_Firmicutes|c_Bacilli|o_Bacillales|f_Staphylococcaceae	CCS	3.8537	0.0015	0.0456
Bacteria|p_Firmicutes|c_Bacilli|o_Bacillales|f_Staphylococcaceae|g_Staphylococcus	CCS	3.8442	0.0015	0.0456
*Bacteria|p_Proteobacteria|c_Gammaproteobacteria|o_Enterobacteriales|f_Enterobacteriaceae|g_*	Control	3.8032	0.0020	0.0456
Bacteria|p_Firmicutes|c_Clostridia|o_Clostridiales|f_Ruminococcaceae|g_Ruminococcus	Control	3.6719	0.0007	0.0397
*Bacteria|p_Bacteroidetes|c_Bacteroidia|o_Bacteroidales|f_Rikenellaceae|g_*	Control	3.5642	0.0020	0.0456
Bacteria|p_Bacteroidetes|c_Bacteroidia|o_Bacteroidales|f_Rikenellaceae	Control	3.5616	0.0020	0.0456
Bacteria|p_Firmicutes|c_Clostridia|o_Clostridiales|f_Lachnospiraceae|g_Lachnospira	Control	3.3193	0.0010	0.0415
Bacteria|p_Proteobacteria|c_Deltaproteobacteria|o_Desulfovibrionales	Control	3.0009	0.0006	0.0397
Bacteria|p_Proteobacteria|c_Deltaproteobacteria	Control	3.0009	0.0006	0.0397
Bacteria|p_Proteobacteria|c_Deltaproteobacteria|o_Desulfovibrionales|f_Desulfovibrionaceae	Control	3.0009	0.0006	0.0397
Bacteria|p_Actinobacteria|c_Actinobacteria|o_Actinomycetales|f_Actinomycetaceae|g_Actinomyces	CCS	2.7365	0.0006	0.0397

Table S2: Taxonomic hierarchy and relative contribution of the sPLS-DA identified group-specific OTUs in the first and second component.

Phylum	Class	Order	Family	Genus	Species	OTU ID	Relative contribution in Survivor group compared to Control group
**OTUs contribution on first component**							
Firmicutes	*Clostridia*	*Clostridiales*	*Ruminococcaceae*			OTU196957	0.6683
Firmicutes	*Clostridia*	*Clostridiales*	*Lachnospiraceae*	*Coprococcus*		OTU1614788	0.3895
Firmicutes	*Clostridia*	*Clostridiales*	*Ruminococcaceae*	*Faecalibacterium*	*Prausnitzii*	OTU199761	0.3497
Firmicutes	*Clostridia*	*Clostridiales*	*Ruminococcaceae*	*Faecalibacterium*	*Prausnitzii*	OTU199145	0.3460
Firmicutes	*Clostridia*	*Clostridiales*	*Ruminococcaceae*	*Faecalibacterium*	*Prausnitzii*	OTU208739	0.3102
Firmicutes	*Clostridia*	*Clostridiales*	*Ruminococcaceae*	*Faecalibacterium*	*Prausnitzii*	OTU199293	0.1815
Firmicutes	*Clostridia*	*Clostridiales*	*[Tissierellaceae]*	*Finegoldia*		OTU12263	-0.1442
Firmicutes	*Clostridia*	*Clostridiales*	*[Tissierellaceae]*	*Peptoniphilus*		OTU494906	-0.0963
Firmicutes	*Clostridia*	*Clostridiales*	*[Tissierellaceae]*	*Peptoniphilus*		OTU654307	-0.0177
Firmicutes	*Clostridia*	*Clostridiales*	*[Tissierellaceae]*	*Peptoniphilus*		OTU4397098	-0.0017
**OTUs contribution on second component**							
Firmicutes	*Clostridia*	*Clostridiales*	*Veillonellaceae*	*Dialister*		OTU258375	0.6203
Firmicutes	*Clostridia*	*Clostridiales*	*Clostridiaceae*	*Clostridium*		OTU95	0.4987
Firmicutes	*Clostridia*	*Clostridiales*	*Lachnospiraceae*			OTU182289	-0.4288
Firmicutes	*Clostridia*	*Clostridiales*	*Clostridiaceae*	*Clostridium*		OTU69664	0.3468
Firmicutes	*Clostridia*	*Clostridiales*	*[Tissierellaceae]*	*Finegoldia*		OTU17111	-0.1775
Firmicutes	*Clostridia*	*Clostridiales*	*Lachnospiraceae*	*Blautia*		OTU289734	-0.1452
Bacteroidetes	*Bacteroidia*	*Bacteroidales*	*Porphyromonadaceae*	*Porphyromonas*		OTU495451	0.0668
Firmicutes	*Clostridia*	*Clostridiales*	*Veillonellaceae*	*Dialister*		OTU57	0.0615
Firmicutes	*Clostridia*	*Clostridiales*	*[Tissierellaceae]*	*ph2*		OTU3289	0.0383
Firmicutes	*Clostridia*	*Clostridiales*	*Veillonellaceae*	*Dialister*		OTU753638	0.0104

Table S3: Partial correlation test, controlling for Age, Gender & BMI.

Taxa		% HLA-DR+ CD4 T cells	% HLA-DR+ CD8 T cells	log(IL6)	log(CRP)
**Data combined from both Survivor and Control groups**					
p_Bacteroidetes	*ρ*	**-0.133**	-0.051	-0.050	**-0.142**
	p-value	**0.009**	0.316	0.350	**0.005**
p_Actinobacteria	*ρ*	**0.216**	**0.221**	-0.028	0.074
	p-value	**0.000**	**0.000**	0.602	0.147
g_Anaerococcus	*ρ*	**0.265**	**0.205**	0.020	**0.232**
	p-value	**0.000**	**0.000**	0.704	**0.000**
g_Finegoldia	*ρ*	**0.225**	**0.294**	**0.111**	**0.117**
	p-value	**0.000**	**0.000**	**0.036**	**0.023**
g_Corynebacterium	*ρ*	**0.159**	**0.223**	**0.108**	0.058
	p-value	**0.002**	**0.000**	**0.043**	0.260
g_Peptoniphilus	*ρ*	**0.148**	**0.190**	**0.175**	**0.202**
	p-value	**0.004**	**0.000**	**0.001**	**0.000**
g_Faecalibacterium	*ρ*	-0.032	-0.073	**-0.211**	**-0.167**
	p-value	0.536	0.151	**0.000**	**0.001**
g_Ruminococcus	*ρ*	0.087	0.041	**-0.213**	**-0.250**
	p-value	0.089	0.425	**0.000**	**0.000**
**Data from Survivor group only**					
p_Bacteroidetes	*ρ*	0.062	-0.021	0.007	0.050
	p-value	0.380	0.763	0.922	0.469
p_Actinobacteria	*ρ*	-0.037	**0.142**	**-0.250**	-0.080
	p-value	0.596	**0.041**	**0.000**	0.247
g_Anaerococcus	*ρ*	0.110	**0.140**	-0.094	0.107
	p-value	0.116	**0.045**	0.191	0.121
g_Finegoldia	*ρ*	**0.192**	**0.474**	-0.026	0.055
	p-value	**0.006**	**0.000**	0.719	0.427
g_Corynebacterium	*ρ*	0.057	**0.269**	-0.100	-0.046
	p-value	0.417	**0.000**	0.164	0.503
g_Peptoniphilus	*ρ*	0.133	**0.316**	0.140	**0.159**
	p-value	0.057	**0.000**	0.052	**0.021**
g_Faecalibacterium	*ρ*	-0.016	-0.096	-0.126	**-0.214**
	p-value	0.815	0.168	0.080	**0.002**
g_Ruminococcus	*ρ*	0.130	0.082	**-0.150**	**-0.226**
	p-value	0.062	0.241	**0.037**	**0.001**

**Data from Control group only**					
p_Bacteroidetes	*ρ*	**-0.235**	0.021	0.006	**-0.247**
	p-value	**0.002**	0.787	0.943	**0.001**
p_Actinobacteria	*ρ*	**0.404**	**0.216**	**0.188**	**0.184**
	p-value	**0.000**	**0.004**	**0.018**	**0.016**
g_Anaerococcus	*ρ*	**0.216**	0.122	0.047	**0.227**
	p-value	**0.004**	0.108	0.561	**0.003**
g_Finegoldia	*ρ*	**0.171**	0.052	0.141	0.051
	p-value	**0.023**	0.492	0.078	0.509
g_Corynebacterium	*ρ*	**0.170**	0.063	**0.279**	0.076
	p-value	**0.024**	0.406	**0.000**	0.326
g_Peptoniphilus	*ρ*	0.077	-0.041	0.150	**0.171**
	p-value	0.307	0.588	0.060	**0.026**
g_Faecalibacterium	*ρ*	0.069	0.091	-0.128	0.061
	p-value	0.360	0.227	0.109	0.433
g_Ruminococcus	*ρ*	**0.248**	**0.193**	-0.060	-0.101
	p-value	**0.001**	**0.010**	0.451	0.190

Note: ρ means correlation coefficient.

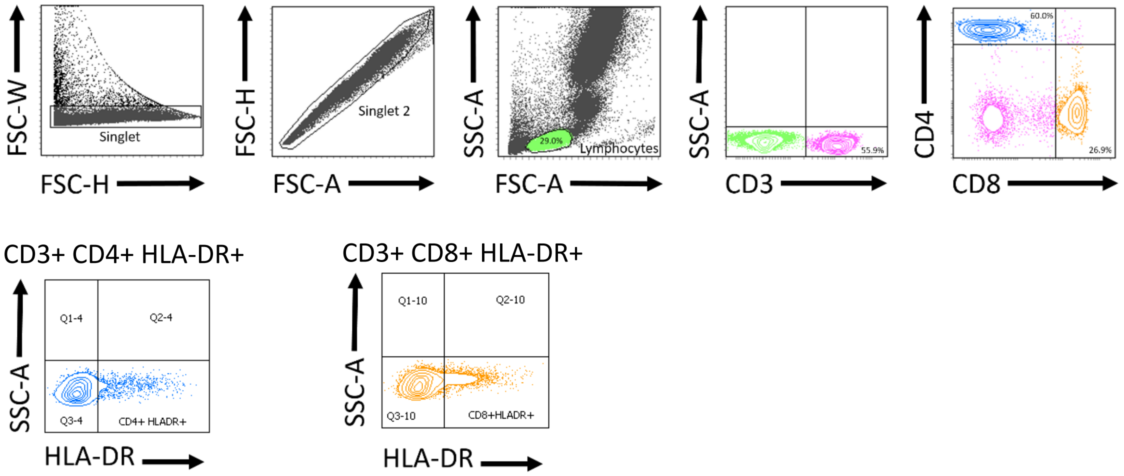

Figure S1. Immunophenotyping of T cell activation

The immunophenotyping was performed on peripheral whole blood as previously described [1]. Samples were acquired on a BD FACSCanto II (BD Biosciences, San Jose, CA) for 100,000 events and analysed using the FACS Diva software (version 6.0). Following gating for doublets exclusion, CD3+ cells were selected and sequentially gated for CD4+ and CD8+ T- cells and subsequently for percentage of activated (HLA-DR+) cells.

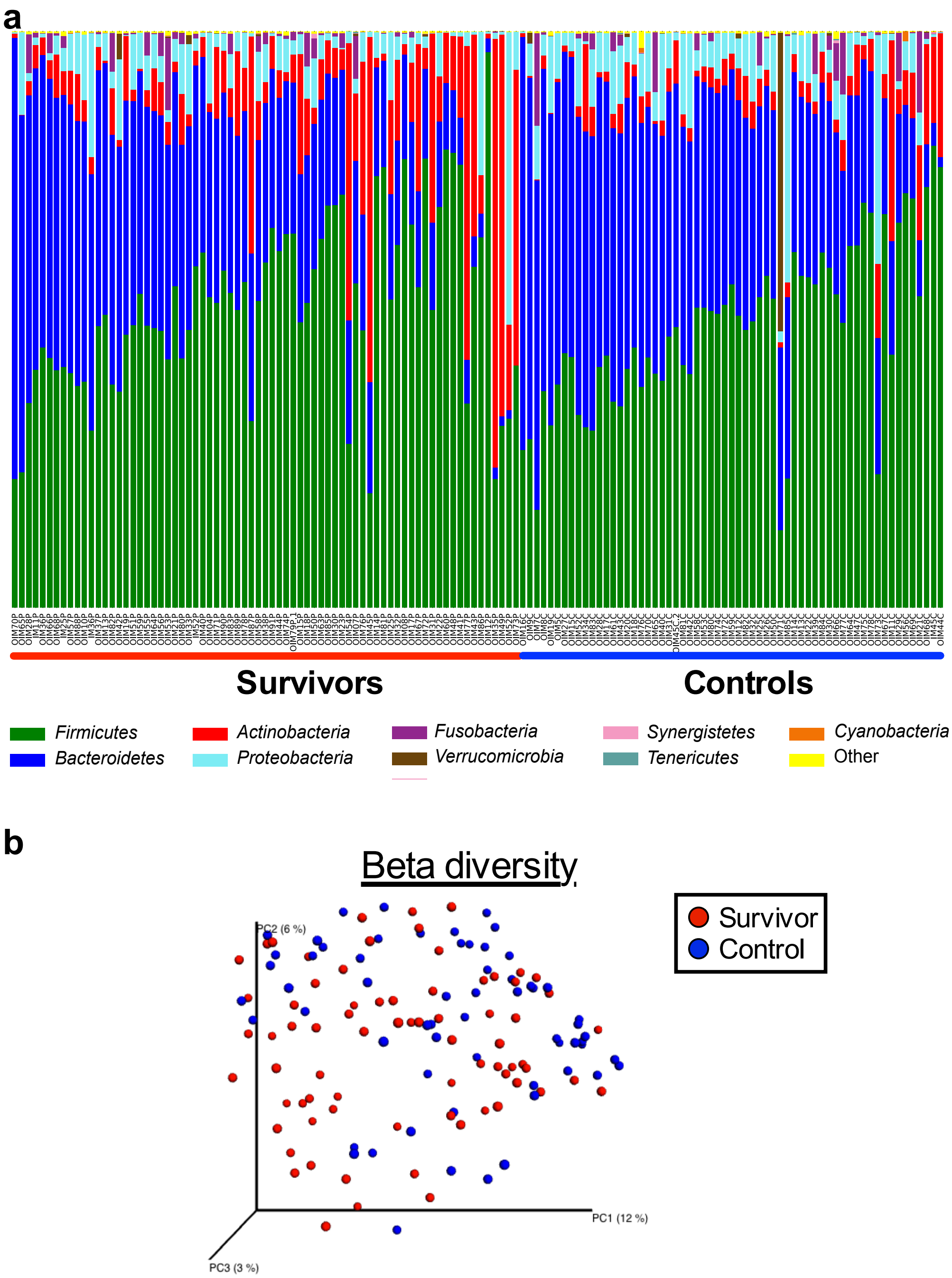

Figure S2. Taxonomy plot and beta diversity

Relative abundance of the phyla represented across the 73 survivors and 61 controls arranged by the abundance of Bacteroidetes from higher to lower (a). Beta diversity of the anal microbial communities in survivor and control samples were calculated with unweighted unifrac distance and displayed on PCoA plot (b).

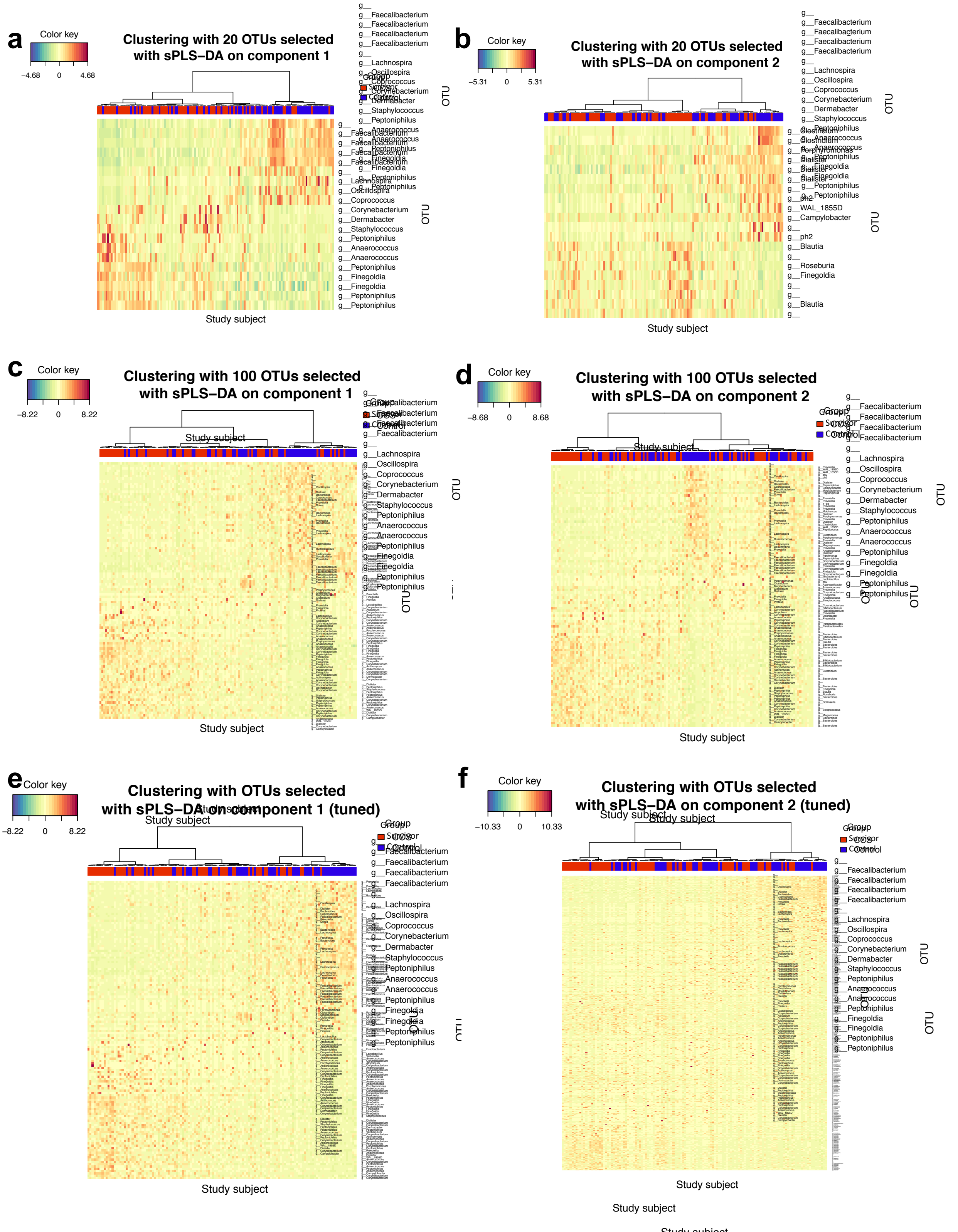

Figure S3. Identification of groups (survivors or controls) associated OTUs using CLR transformation and sPLS-DA model with a threshold set at a different number of OTUs. The abundance of each selected OTUs are presented on clustering heatmaps for sPLS-DA model with 20 OTUs selected (a and b); with 100 OTUs selected (c and d) and with the optimal number of OTUs identified through the MixOmics tuning process. 123 OTUs were selected for component 1 while 407 were selected for component 2.

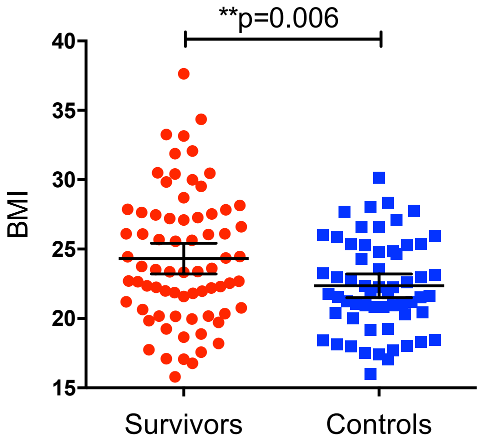

Figure S4. Body mass index (BMI) between adult survivors of childhood ALL and controls. Survivor group has higher mean BMI as compared to the control group. Data shown as mean ± 95% confident interval and P-values was calculated by the parametric Student T test.

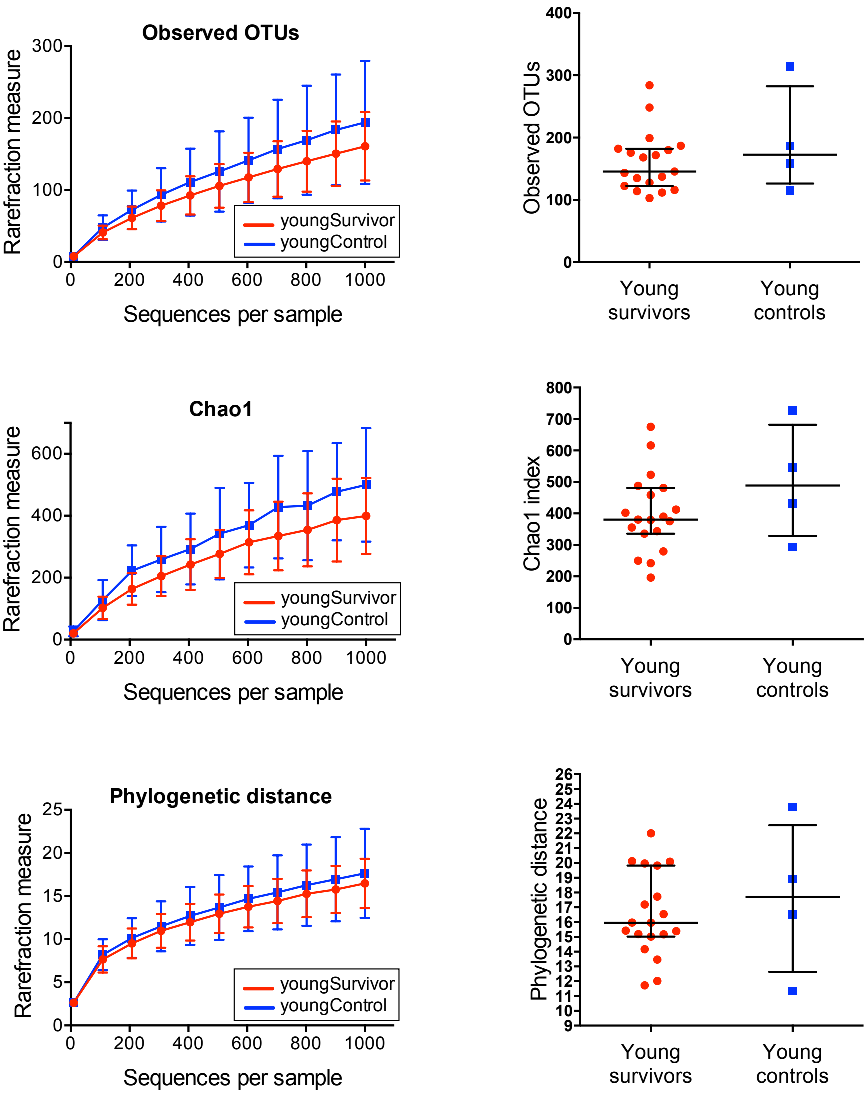

Figure S5: Reduced microbial diversity in young (9-17 years old) survivors of childhood ALL. Alpha diversity measured with three different matrices: Chao1, phylogenetic distance and observed OTUs. Microbial diversity has a trend of reduction among young survivors (n= 19) as compared to young controls (n=4), but statistical test was not performed due to the small sample size of young controls in this cohort. Young survivors and controls are defined by age less than18 years old. OTUs, operational taxonomic units.

References

1. Azanan MS, Abdullah NK, Chua LL, Lum SH, Abdul Ghafar SS, Kamarulzaman A, et al. Immunity in young adult survivors of childhood leukemia is similar to the elderly rather than age-matched controls: Role of cytomegalovirus. Eur J Immunol 2016;46(7):1715-26.
